# Women’s Preferences of Method of Delivery and Influencing Factors

**DOI:** 10.5812/ircmj.11532

**Published:** 2013-08-05

**Authors:** Sema Dereli Yilmaz, Meltem Demirgoz Bal, Nezihe Kizilkaya Beji, Seyfettin Uludag

**Affiliations:** 1 Department of Midwifery, Health Sciences Faculty of Selcuk University, Konya, Turkey; 2 Department of Gynecologic and Obstetrics Nursing, Health College of Karamanoglu Mehmetbey University, Karaman, Turkey; 3 Department of Gynecologic and Obstetrics Nursing, Florence Nightingale Nursing Faculty of Istanbul University, Istanbul, Turkey; 4 Department of Obstetrics and Gynecology, Cerrahpasa Medical School of Istanbul University, Istanbul, Turkey

**Keywords:** Caesarean Section, Vaginal Birth after Cesarean, Pregnancy

## Abstract

**Background:**

Currently, the rate of caesarean section has been substantially increased in developing and developed countries. To determine the factors causing such an increase, it is important to determine reasons for women to refuse vaginal delivery and preferring caesarean section.

**Objectives:**

To determine Turkish women’s attitudes and basal knowledge regarding vaginal delivery and caesarean section, as well as factors causing women to prefer caesarean section even when a medical indication does not exist.

**Patients and Methods:**

This descriptive study consisted of 840 women, completing the questionnaire developed by the researchers.

**Results:**

Mean age rate of participants was 39.8 ± 11.8 years. The most significant reasons of vaginal delivery preferred by participants (n = 685) were determined to be healthy and swift recovery period after delivery, whereas those preferred by participants (n=155) for caesarean section were being safer for babies, easier than vaginal delivery and a less painful method. Higher educational status, pregnancy after infertility treatment and undergoing caesarean section for the last delivery were determined to be among important factors affecting to choose caesarean section.

**Conclusions:**

Information gained misleadingly and fears related to vaginal delivery were seen as factors affecting women’s preferences for delivery. Thus, midwives are required to train both pregnant women during antenatal care and all women in society about methods of delivery and to give effective counseling.

## 1. Background

Despite a surgical procedure that is performed to protect maternal and foetal health, caesarean section (CS) has recently become a delivery method preferred by expectant mothers beyond a medical or obstetric modality ordered by specialists, if necessary. Reported to be increased in developing and developed countries, the rate of CS was found to be just 5% in 1970s, to elevate a quarter of deliveries in 1988, to be 24.5% in 2001 and to ascend up to 32% in 2007 in the USA (1-4). In Canada, the rate was increased to 22% between 2000 and 2001, while 18% between 1994 and 1995 (5). The rate of CS in Great Britain was 12.5% in 1990, while increasing up to 18.3% in 1999 (6). Under the criteria of the World Health Organization, the rate of CS should not be allowed to exceed 15%; however, this rate has so far been exceeded far more than recommended levels in many developing countries, including Turkey (37%) (7). Reasons should be identified to develop strategies for the prevention of such an increment.

While deciding the type of delivery, most women feel under stress and are affected by such factors as fears, anxiety and sympathy as well as logical approach. Along with such factors, perception of delivery in society where expectant mothers live, socio-demographic and psychosocial features, and effects from members of family and friends are among other traits influencing the process (8). In developing countries like Turkey, social norms are passed on from generation to generation, so such norms are influential factors on the preferences of delivery by women. Expectant mothers obtain information related to delivery, especially from members of family, friends, the environment in which they live, and the process may be affected by their experiences, recommendations and social sanctions. Thus, it is significant to define the tendency of women related to the type of delivery.

Lowering the rate of CS on maternal request and encouraging vaginal delivery (VD) are required for maternal and foetal health. Thus, it is of great importance to identify the reasons affecting preferences of delivery. In literature, studies investigating women’s preferences of delivery that are pregnant or in postpartum period are present (9, 10); however, studies evaluating the general tendency of such women, level of information and preferences of women in society were rarely encountered.

## 2. Objectives

The aim of our study is to determine the basal knowledge of women giving at least one birth over the types of delivery and the factors affecting the preference of delivery.

## 3. Patients and Methods

The present descriptive study was conducted at Gynecology and Obstetrics Department of Cerrahpasa Medical Faculty of Istanbul University between March and August 2011. The study was approved by the Ethics Committee of the institution. Women consecutively admitted to the clinic for examination were informed about the design and aim of the study, and those with at least one birth, no difficulty in communication and accepting to participate in the study were included. Those with previous CS due to medical requirements were excluded out of the study. A questionnaire formed by researchers was given to each participant, and all participants were asked to complete questionnaires in a special chamber. The data were accumulated via the questionnaire including socio-demographic and obstetric characteristics of participants, and determining women’s preferences for delivery.

Three experts were consulted to determine whether the items in the questionnaire had been prepared as consistent with the aim of the study, and in light of their recommendations, the last version was formed. A pilot study was carried out with 15 women to find out the understandability of the questionnaire, and no data obtained from the pilot study were used in the following process. While calculating sample size, the birth rate in Turkey related to CS was considered to be 37%, a datum obtained from Turkey Demographic and Health Survey (TDHS) (7). The sample size of 861 was calculated by considering 37% via G*Power 3.1 to indicate with power of 0.85 in difference of 5% (11).

In the analysis of data, Statistical Package for the Social Sciences (SPSS, the version of 20.0) was used. Data were represented as number and percentage. In the statistical analysis of the data, chi-square test, Fisher’s exact test and logistic regression analysis were performed. P values less than 0.05 were accepted to be statistically significant. Logistic regression analysis was used to determine the factors effective on women’s preference of CS in general and as the last delivery.

## 4. Results

Given that losses might occur while collecting data, 870 women were interviewed, and seven rejected to participate in the study. Eight hundred and sixty-three women were delivered questionnaires, and when the questionnaires were collected, 23 women failed to fill in the questionnaires and were excluded due to incomplete data. So, the analyses could be performed with 840 women. Of the women, 564 were determined to experience VD and 276 to experience CS. Mean age rate of participants was 39.8 ± 11.8 years, mean duration of marriage was 18.0 ± 12.5 years, mean level of education was 7.8 ± 3.7 years, mean number of pregnancies was 3.3 ± 2.2, mean number of births was 2.4 ± 1.6, mean number of spontaneous abortions was 1.7 ± 1.1, and 82% were unemployed. Among the women, 80% were followed-up by a physician in the course of their pregnancies, 24% were informed about VD, 20% were informed about CS by a healthcare professional, and 73% were assisted by a specialist during the delivery. The last delivery was vaginally performed by 67% of all participants (n = 564), whereas the rate was 33% among those giving the last birth via CS (n = 276). Within all participants, 90.2% of those giving the last birth vaginally and 36.2% of those giving the last birth via CS reported to prefer the following birth in the same way. When all participants were asked whether to prefer VD or CS, 81.5% and 18.5% reported that they would prefer VD (n = 685) and CS (n = 155), respectively. When the basal knowledge of women preferring CS was questioned, the reasons why CS was preferred were determined to be the fear of labour pain, finding CS as less painful, easier for mother and safer for mother and baby, with no vaginal damage, and no risk for urinary incontinence (Table 1). 

**Table 1.** Basal Knowledge and Reasons of Women’s Preference Related to Caesarean Section

	Yes, no. (%)	No, no. (%)
**I prefer CS** **a** **due to the fear of labour pain**	126 (81.3)	29 (18.7)
**CS is a less painful method**	122 (78.7)	33 (3.9)
**CS is easier than VD^a^**	111 (71.6)	44 (28.4)
**CS causes no vaginal injury**	104 (67.1)	51 (32.9)
**CS is safer for baby**	92 (59.4)	63 (40.6)
**CS is performed in more sterile conditions**	77 (49.7)	78 (50.3)
**Risk of urinary incontinence is present after VD**	72 (46.5)	83 (53.5)
**CS may provide the control of birth date**	61 (39.4)	94 (60.6)
**CS is safer for mother**	60 (38.7)	95 (61.3)
**CS has no influence on postpartum sexual life**	57 (36.8)	98 (63.2)
**I prefer CS due to negative effects of previous VD**	55 (35.5)	100 (64.5)
**CS is a more modern method**	54 (34.8)	101 (65.2)
**I prefer CS due to recommendations by physicians or midwives**	49 (31.6)	106 (68.4)
**I prefer CS feeling under the effects of mass media instruments**	6 (3.9)	149 (96.1)

^a^ Abbreviations: VD, Vaginal delivery; CS, Caesarean section

On the other hand, women reported that they preferred VD due to being informed previously about VD, and such reasons as feeling of self-control during the delivery, finding it healthy and natural, more comfortable postpartum period, breastfeeding the baby earlier, swift healing process, no exposure to anaesthetics, seeing it as a safer approach to both mother and baby and experiencing previous VD (Table 2). 

**Table 2.** Basal Knowledge and Reasons of Women’s Preference Related to Vaginal Delivery

	Yes, no. (%)	No, no. (%)
**I prefer VD** ^**a**^ **for being healthy**	611 (89.2)	74 (10.8)
**Due to swift postpartum recovery**	607 (88.6)	78 (11.4)
**VD has more comfortable postpartum period**	564 (82.3)	121 (17.7)
**VD is natural**	517 (75.5)	168 (24.5)
**VD gives a chance for early breastfeeding**	506 (73.9)	179 (26.1)
**VD is safer for mother**	465 (67.9)	220 (32.1)
**I prefer VD due to previous experience**	446 (65.1)	239 (34.9)
**I prefer VD due to not being exposed to anaesthesia**	424 (61.9)	261 (38.1)
**VD is safer for baby**	409 (59.6)	276 (40.4)
**VD provides self-control**	407 (59.4)	278 (40.6)
**VD provides shorter pain period than CS^a^**	457 (54.4)	228 (27.1)
**I prefer VD due to recommendations by physicians or midwives**	146 (21.3)	539 (78.7)

^a^ Abbreviations: VD, Vaginal delivery; CS, Caesarean section

Among the factors associated with women’s choice on type of delivery, educational status, level of income, healthcare providers following women in the course of pregnancy, pregnancy following infertility treatment and type of last delivery were found to be statistically significant (P < 0.05) (Table 3). 

**Table 3.** Factors Associated With Women’s Preferences of Type of Delivery and the Last Delivery

	Preferred Method of Delivery			Type of the Last Delivery		
	VD^a^(n = 685)	CS^a^(n = 155)	p	VD (n = 564)	CS (n = 276)	P
**Age**						
≤ 30 years	191	34	0.131	129	96	< 0.001
≥ 31 years	494	121		435	180	
**Educational status**						
≤ 8 years	509	77	< 0.001	440	146	< 0.001
≥ 9 years	176	78		124	130	
**Occupational status**						
Employed	119	36	0.090	84	71	< 0.001
Unemployed	566	119		480	205	
**Level of income**						
Income lower than expenditure	331	91	0.019	307	115	0.001
Income equal to/higher than expenditure	354	64		257	161	
**Type of health professional following-up during pregnancy**						
Midwife	160	12	< 0.001	170	2	< 0.001
Physician	525	143		394	274	
**Number of births**						
1	224	54	0.609	147	131	< 0.001
≥ 2	461	101		417	145	
**Pregnancy following infertility treatment**						
Yes	21	23	< 0.001	10	34	< 0.001
No	664	132		554	242	
**Being informed about VD**						
Yes	161	41	0.438	123	79	0.030
No	524	114		441	197	
**Being informed about CS**						
Yes	136	34	0.560	87	83	< 0.001
No	549	121		477	193	
**Type of the last delivery**						
VD	509	55	< 0.001			
CS	176	100				

^a^ Abbreviations: VD, Vaginal delivery; CS, Caesarean section

Regression analysis was performed to determine to what extend these factors were effective in the women’s preference of CS. Logistic regression analysis revealed that educational status of ≥ 9 years (OR=2.9), pregnancy following infertility treatment (OR = 3.8) and undergoing CS for the last delivery (OR=4.8) increased the risk for preferring CS, whereas level of income equal or higher than expenditure (OR=0.4), being at the age of ≤ 30 years (OR = 0.6) and primiparity (OR = 0.6) decreased the risk (Table 4). 

**Table 4.** Logistic Regression Analysis for Factors Affecting Women’s Preference of Caesarean Section (Enter Method)

Independent Variables^a^	B	OR	95% CI	P^b^
**≤ 30 years**	-0.510	0.601	0.370-0.975	0.039
**Educational status of ≥ 9 years**	1.078	2.939	1.841-4.691	< 0.001
**Income equal to/higher than expenditure**	-0.919	0.399	0.263-0.606	< 0.001
**Primiparity**	-0.626	0.535	0.343-0.834	0.006
**Pregnancy following infertility treatment**	1.323	3.754	1.877-7.510	< 0.001
**Undergoing CS for the last delivery** ^**c**^ ** for the last delivery**	1.575	4.830	3.105-7.512	< 0.001

^a^Reference data of Logistic regression analysis.

^b^To evaluate common effect of independent variables, all independent variables presented in Table 3 were included into the model, and those of P < 0.05 were indicated in Table 4.

^c^ Abbreviations CS: Caesarean Section

Age, educational and occupational status, level of income, healthcare providers following-up the pregnancy, number of births, pregnancy following infertility treatment, being informed about VD and CS and preferred method of delivery were found to be significantly associated with the type of the last delivery (P < 0.05) (Table 3). To the results of logistic regression analysis, educational level of ≥ 9 years (OR = 1.6), level of income equal or higher than expenditure (OR = 1.5), being followed-up by a physician during pregnancy (OR = 36.5), primiparity (OR = 1.7), pregnancy following infertility treatment (OR = 2.8), lack of information about VD (OR = 6.9), being informed about CS (OR = 9.7) and preferring CS as the method of delivery (OR = 4.8) were found to increase the risk for women to give the last birth as CS (Table 5). 

**Table 5.** Logistic Regression Analysis for Factors Affecting Women’s Preferences of Caesarean Section as Method of the Last Delivery (Enter Method)

Independent Variables^a^	B	OR	95% CI	P^b^
**Educational status of ≥ 9 years**	0.500	1.648	1.098-2.474	0.016
**Income equal to/higher than expenditure**	0.384	1.468	1.023-2.105	0.037
**Being followed-up by a physician during pregnancy**	3.596	36.449	8.858-149.982	< 0.001
**Primiparity**	0.549	1.731	1.185-2.530	0.005
**Pregnancy following infertility treatment**	1.039	2.826	1.286-6.211	0.010
**Being uninformed about VD** ^**c**^	1.927	6.871	2.755-17.132	< 0.001
**Being informed about CS^c^**	2.273	9.712	3.822-24.682	< 0.001
**Preferring CS as a method of delivery**	1.565	4.822	3.085-7.537	< 0.001

^a^Reference data of Logistic regression analysis

^b^To evaluate common effect of independent variables, all independent variables presented in Table 3 were included into the model, and those of p < 0.05 were indicated in Table 5.

^c^ Abbreviations VD: Vaginal delivery; CS: Caesarean section

## 5. Discussion

Among women, individual trust and expectations concerning birth might change from person to person. Experiences of other women may also be influential on women’s preferences of method of delivery, as well as expectations of mothers for themselves and their babies. Therefore, it is important to reveal women’s expectations from childbirth and to determine women’s basal knowledge, preferences and related factors to the type of delivery in order to give necessary information, support and care in this process. In fact, type of previous delivery may be an important determinant influencing on women’s preferences of delivery. In our study, 90% of women giving the last birth through vaginal route and 36% of those giving the last birth by CS reported that they would prefer the same method. As different from our findings, in a study, 23.8% of 259 women with VD were determined to prefer CS after the first delivery, and only 5 of 25 women with elective CS changed their mind to prefer VD (10). Our findings indicated that the number of women preferring VD is higher in Turkey, compared to those found in the study by Pang et al. (10).

In the present study, women preferring VD were of the opinion that VD was a healthier and more natural way of giving birth, and these factors were being followed by more comfortable postpartum period and swift postpartum recovery, compared to CS. In a study in which pregnant women of 37 weeks reporting to prefer VD were questioned six months after the delivery about how they would prefer the next delivery and related factors, the women were determined to prefer VD again due to swift postpartum recovery (27.5%) and being natural (24%) (10). In the study conducted by Pevzner et al. (12), it was detected that of the participants, 93% found VD as healthy for mothers, 88% as healthy for babies, more than a third (34%) found natural route to be better, 29% saw CS as a risk for complications, and 13% asserted that CS should only be performed in the presence of a risk. Seventy-six percent of women in a study by Dursun et al. (13) and 61.9% of those in our study stated that VD did not indicate a risk for anaesthesia and operation. In the study (13), more than half of women (59%) reported that babies given birth via vaginal route were healthier. Likewise, 67.9% of our participants were of the opinion that VD was safer for mothers, and to 59.6%, it was safer for babies. In our study, 73.9% of women reported that they preferred VD due to early breastfeeding. As seen in different studies, women uttered the common advantages of VD. Therefore, it is important for healthcare professionals to train and inform women about the other unknown advantages of VD, emphasizing its importance.

While the rate of CS is lower in third world countries due to shortage of medical facilities, the rate is increased in developed countries and getting increased in developing ones (1-4). According to the data from TDHS, the rate of CS in Turkey, a developing country, increased from 21% in 2003 to 37% in 2008 (7). In our study, elective CS was preferred by 18.5% of women, and the same rate was detected in various studies as follows: 15.9% in the study conducted by Buyukbayrak et al. (9), 3.7% in Singapore (14), 8.7% in Sweden and 18.2% in Australia (15). It is seen that the more elective CS is preferred in a country, the more the rate is also increased in that country. Thus, midwives should definitely train and give information to women over health benefits of VD while providing healthcare services related to obstetrics and gynaecology, because CS can be a method to be performed only in the presence of an obstetric indication.

As consistent with our findings, in another study by Sercekus and Okumus (16), the most common source of fear related to childbirth was reported to be labour pain, and the participants in their study reported to be afraid of failure to give birth and losing their control. In the study by Ryding (17), it was determined that women usually preferred CS because of concerns about labour pain and health of babies. While another study carried out in Turkey stated that majority of women preferred CS due to fear of VD, a fifth of the participants was reported to prefer CS due to finding it safer for babies (9). In a study in Singapore, it was also found that more than half of the women preferred CS because of avoidance of labour and stress (14). In the study conducted by Fenwick et al. (18), among main factors affecting women’s choice of delivery as elective CS were concerns related to control and safety, fear of VD, seeing women’s bodies as deformed due to childbirths and birth process. In light of these studies, women and their partners should be trained and informed during antenatal care in favour of VD to cope with labour pain.

Within our participants, 67.1 and 36.8% had knowledge about CS not to cause vaginal injuries and not to affect sexual life negatively, respectively. Likewise, in a study, it was determined that women were worried, because VD could give harm to vagina and aesthetic appearance of genital area (16). Nearly half of our participants and 33% of women in the study by Dursun et al. (13) reported to have knowledge about the risk of developing urinary incontinence after VD. Such concerns are considered to be widespread among women and be eliminated by effective training sessions provided by midwifes.

Another factor leading to an increase in CS is also educational status. In parallel to our findings, many studies are present in literature showing that the rate of CS is increased as educational status increases (19-21). In a study performed in rural parts of China, receiving antenatal care was identified to be a factor increasing the rate of CS (19). A study conducted by The American College of Obstetricians and Gynaecologists on 600 obstetricians and gynaecologists reported that more than half of the participants (53%) performed CS on maternal request (22). In a study conducted in eight European countries to assess the attitudes of obstetricians, the rate of CS on maternal request was detected to change between 15% and 79% (23). As consistent with our findings, according to the data from TDHS-2008, women were reported to begin receiving antenatal care in recent years from physicians rather than midwives. Accordingly, the rate of CS was reported to be increased in Turkey (7). We consider that physicians manipulate women to CS with the opinion of the time spent for VD that will last longer during the follow-up, and that women planning CS at the very beginning choose to get antenatal care from physicians. In a study performed by Gozukara and Eroglu (24), the rate of CS was determined to be higher in those with infertility treatment, and it was consistent with our findings. Likewise, Basso and Baird (25) reported that the rates of both acute and planned CS were higher among pregnant women with infertility treatment, compared to those with spontaneous pregnancies. It may be considered that the couples trying to get pregnant for a long time might have found VD as a risk for both maternal and foetal health. Moreover, the fact that assisted reproductive technologies increase the rate of multiple pregnancies might also be considered a reason directing both physicians and families to CS.

The present study was conducted in Istanbul, an immigration province from all regions of Turkey, with the highest population in the country. Therefore, the sample size of our study is quite rich both quantitatively and qualitatively due to the performance of the study in a university hospital receiving patients from all socioeconomic layers. Our study may be seen as intriguing in terms of basal knowledge of women on delivery methods and the determination of their preferences. Moreover, it is remarkable that women are also lack of accurate knowledge about VD and CS. So, women are recommended to be trained and well-informed by midwives about VD and CS.
